# P-694. Patient Specific Predictors of Mortality in Treatment of Community Acquired Pneumonia: A Retrospective Analysis

**DOI:** 10.1093/ofid/ofaf695.907

**Published:** 2026-01-11

**Authors:** Saher Siddiqui, Maria Santana-Garces, Marcus Zervos, Jamie G Joseph, Anqi Wang

**Affiliations:** Henry Ford Health, West Bloomfield, MI; Henry Ford Health, West Bloomfield, MI; Henry Ford Hospital, Detroit, Michigan; Henry Ford Health/MSU, Royal Oak, Michigan; Henry Ford Health, West Bloomfield, MI

## Abstract

**Background:**

Pneumonia is a leading cause of morbidity and mortality in hospitalized adults. Prompt identification of high-risk patients and initiation of therapy is important in improving outcomes in community acquired pneumonia (CAP). This study evaluates predictors of 30-day mortality anywhere in hospitalized CAP patients.

**Table 1:**
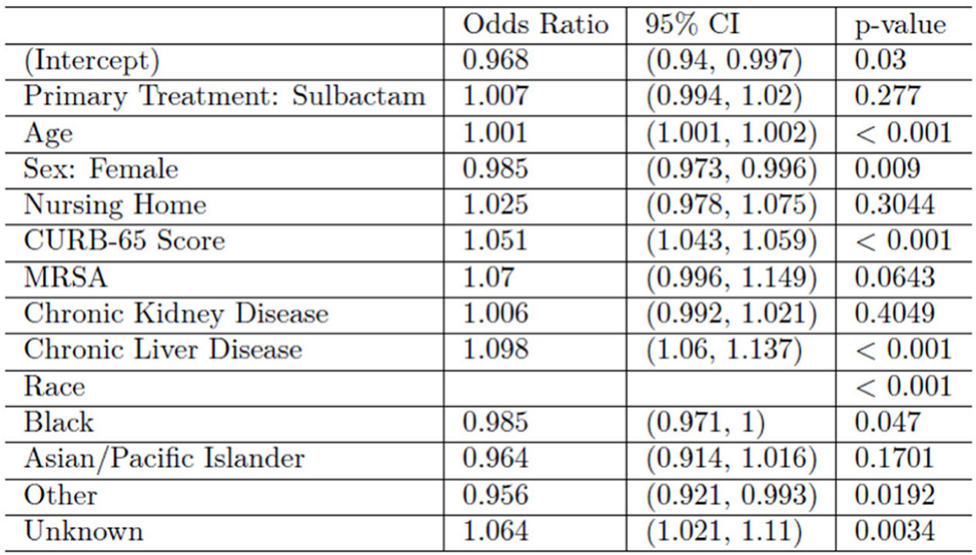
30-Day Mortality Regression Results Logisitic regression was performed on unmatched cohort

**Methods:**

This was a retrospective cohort study of n=10,640 hospitalized adults diagnosed with CAP between January 2022 and August 2024 at Henry Ford Hospital. Data regarding age, sex, race, nursing home residency, history of chronic kidney disease (CKD), history of chronic liver disease (CLD), history of methicillin-resistant Staphylococcus aureus (MRSA), and pneumonia severity (using CURB-65 score) was collected for each patient. Logistic regression was used to test the association between various clinical and demographic characteristics and 30-day mortality.

**Results:**

Patients with increased CURB-65 score had higher odds of death within 30 days (anywhere/in-hospital) aOR= 1.05 (95% CI 1.043-1.059, p= < 0.001). Other factors that resulted in increased odds of 30-day mortality include increasing age (OR 1.001, 95% CI 1.001-1.002, p= < 0.001) and presence of CLD (aOR 1.098, 95% CI 1.06-1.137, p=< 0.001). The adjusted odds ratio comparing odds of 30-day mortality between female and male patients was 0.985; female patients had significantly lower odds of 30-day mortality (95% CI 0.973-0.996, p=0.009).

**Conclusion:**

30-day mortality was significantly associated with clinical factors, including age, sex, and illness severity determined by using the CURB-65 score. These findings largely align with past literature, with the exception of female sex being associated with lower odds of death. Future work should further investigate clinical and demographic factors associated with mortality for patients with CAP.

**Disclosures:**

Marcus Zervos, MD, merck: Honoraria

